# Angiographic Embolization with Histoacryl in Combination with Direct Injection of Bone Cement of an Intraosseous Venous Malformation of the Mandible: Report of a Case with 22-Year Follow-Up

**DOI:** 10.1155/2022/6842968

**Published:** 2022-02-16

**Authors:** Antoine Berberi, Georges Aoun, Georges Aad, Saad Khairallah, Ghassan Abi Chedid

**Affiliations:** ^1^Department of Oral and Maxillofacial Surgery, Faculty of Dental Medicine, Lebanese University, Lebanon; ^2^Department of Oral Medicine and Maxillofacial Radiology, Faculty of Dental Medicine, Lebanese University, Lebanon; ^3^Department of Pathology, Faculty of Medicine, Lebanese University and Director Institute National de Pathology, Lebanon; ^4^Department of Radiology, Sacre-Coeur Hospital, Baada Lebanon and Head Department of Medical Imaging, Lebanese German University, Beirut, Lebanon

## Abstract

Vascular malformations of the maxillofacial region are unusual, and they occur more rarely in bone than in soft tissue. Mandibular intraosseous vascular lesions represent 0.5-1.0% of all bone tumors, and they are classified as venous malformation, lymphatic malformation, arterial malformation, arteriovenous malformations, and arteriovenous fistulae. Venous malformation is the most common vascular malformation, accounting for 44-64% of all vascular malformations, and is considered a low-flow malformation. Endovascular therapy as selective angiographic embolization is considered as the first-choice treatment associated or not with emboli injections with a success rate of 70%, and this evades mutilating surgery and related sequelae. We report a case of mandibular venous malformation on a 45-year-old female complaining of unilateral swelling of the left body of the mandible with facial deformation. The computed tomography scan images and the T1-weighted MR images showed a lesion that expresses an expansible lesion in the spongy bone of the left of the mandible with a buccal cortical rupture. Signal voids were not identified, suggesting a low-flow vascular lesion. The T2-weighted images exposed hypersignals; accordingly, a vascular lesion was suspected. The treatment was done under locoregional analgesia; after selective angiography, direct histoacryl injection was completed, followed by bone cement injection. The patient was followed yearly since1998. Radiological images of 10-year follow-up MRI showed a stabilization of the lesion without any new extensions. The panoramic radiograph after 22 years showed a bone formation inside the body of the mandible. The long follow-up period and the absence of any complications are favorable for the adopted treatment plan.

## 1. Introduction

Vascular lesions were considered as hemangiomas before the1980s, and it was in 1982 that Mulliken and Glowacki proposed a classification for vascular anomalies based on pathological features [1]. Afterwards, the International Society for the Study of Vascular Anomalies (ISSVA) updated the old classification and splits vascular anomalies into two categories: vasoproliferative neoplasms (VN) and vascular malformations (VM) [2].

VMs include capillary malformation (CM), venous malformation (VM) (intraosseous vascular malformation), lymphatic malformation (LM), arterial malformation (AM), arteriovenous malformations (AVM), and arteriovenous fistulae (AVF) [2].

In addition, VMs can be divided based on the degree of blood flow: “slow flow” (CM, VM, or LM), “fast flow” (AM, AVM, or AVF), and combined vascular malformations [3].

VMs are the most common vascular malformation, accounting for 44-64% of all vascular malformations, and are considered as low-flow malformations [4].

Management of VMs is very complex and needs a multidisciplinary team for successful results [5]. At present, super-selective angiographic embolization is considered as the first-choice treatment [6, 7], in association with injectable embolic [8–10].

We describe a case of intraosseous multilocular VM of the mandible where a conservative treatment was applied with a follow-up period of 22 years.

## 2. Case Report

A 45-year-old female was oriented to our department complaining of unilateral swelling of the left body of the mandible with facial deformation.

**Figure 1:**
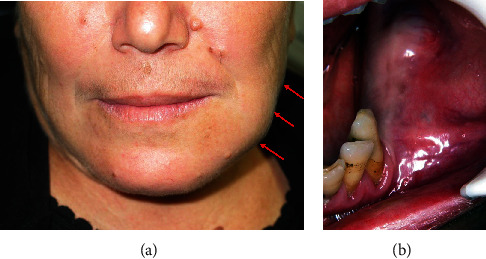
(a) Facial deformation in regard with the left mandible. (b) Tumefaction filling the buccal posterior area of the mandible.

Clinical exam revealed a well-delimited hard swelling at the left of the mandible (body and angle) with submandibular adenopathy. Intraorally, a hard tumefaction that fills the buccal posterior area of the mandible was noticed. Mobility scale 3 of the second premolar was observed, and no paresthesia was noted (Figures 1(a) and 1(b)).

History reveals that the swelling started three years ago without any pain, and the left mandibular first and second molars were extracted as they were mobile without any complications or hemorrhage.

**Figure 2:**
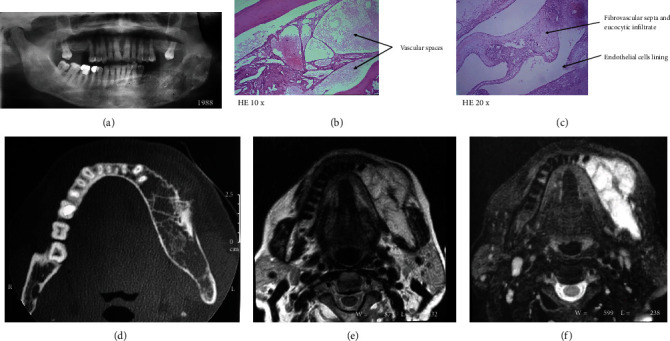
(a) Panoramic radiograph showing a well-defined multilocular radiolucency extending from the mental nerve region to the ramus. (b) Thin-walled and dilated vascular spaces of variable diameter (HE ×10). (c) Regular endothelial lining of the vascular spaces with mild inflammatory polymorphous leucocytic infiltrates in the surrounding tissue (HE ×20). (d) An axial CT image showing an expansible multilocular radiolucency lesion with periosteal reaction on the buccal cortex. (e) An axial T2WI MR image revealing a hyposignal intensity expansible low-intensity lesion in the marrow of the left hemimandible with cortical rupture and soft tissue extension. (f) An axial T2 WI FSE image presented a hypersignal and expansible lesion with internal loculations suggesting a multilocular lesion.

A 2D-panoramic radiograph showed then a well-defined multilocular radiolucency, spreading from the left mandibular mental nerve region anteriorly to the ramus posteriorly; small loculations and fine trabeculae were noted inside the lesion (Figure 2(a)).

Blood test analysis was performed, and the results were in the range of normality (bleeding time: 2 min; blood platelet count: 322 K/*μ*L).

The differential diagnosis varies between ameloblastoma, odontogenic myxoma, giant-cell granuloma, fibrous dysplasia, aneurysmal cyst, and vascular malformations.

After concertation with the patient and her family, the mobile second premolar was extracted and a biopsy of the lesion was taken through the alveolar socket. No hemorrhage was noted, but for safety, a Gelfoam® sponge was placed in the alveolar socket, stabilized with resorbable sutures, and covered with cyanoacrylate tissue glue.

The histological findings of the biopsy (×10 with hematoxylin and eosin coloration) showed a single layer of endothelial cells lining the dilated vascular spaces with mild inflammatory polymorphous leucocytic infiltrates in the surrounding tissue with thin-walled and dilated vascular spaces of variable diameter (Figures 2(b) and 2(c)).

Following the clinical, radiological, and histological results, a CT scan and MRI were requested to determine the soft and hard tissue extensions of the lesions.

### 2.1. CT Scan and MRI Examinations

The CT scan images presented an expansible multilocular lesion with a distinct periosteum reaction on the buccal cortical bone. No loculations were detected, and the axial images displayed a trabeculated lesion in the body of the left mandible (Figure 2(d)).

MRI images with 1.5 T using routine T1- and T2-weighted spin-echo sequences showed the existence of a lesion of the entire left part of the body of the mandible and extending to the ramus without reaching the condyle.

The T1-weighted MR images showed a lesion with hyposignals that express an expansible lesion in the spongy bone of the left of the mandible with a buccal cortical rupture. Signal voids were not identified, suggesting a low-flow vascular lesion (Figure 2(e)).

The T2-weighted images exposed hypersignals; accordingly, a vascular lesion was suspected. Axial cuts displayed high signal intensity and an expansible lesion in the bone marrow of the left hemimandible, with a buccal bone discontinuity and extension towards the soft parts of the vestibular edge of the mandible, producing a subcutaneous tumefaction. Inner loculations were present in the axial cuts suggesting multilocularity (Figure 2(f)).

Clinical, radiological, and histological findings leaded to a conservative treatment, based on embolization and bone cement injections. The treatment plan was suggested and discussed with the patient and her family who signed a consent form.

### 2.2. Angiography/Embolization/Bone Cement Injection

Angiography, through a transfemoral approach, was achieved under local anesthesia in the hospital, and selective arteriography of the left external carotid artery was performed.

**Figure 3:**
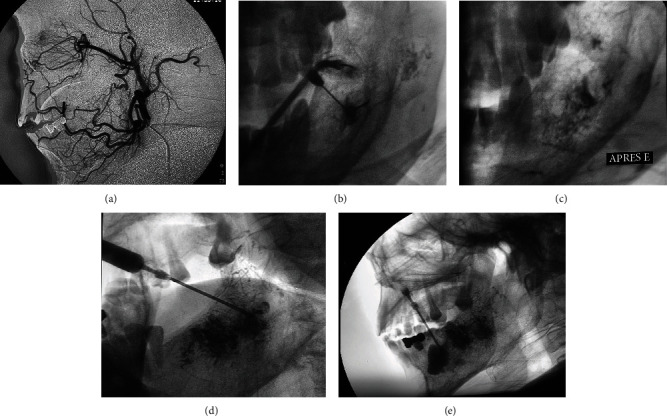
(a) Lateral digital subtraction angiography image displayed the inferior alveolar and lingual arteries. (b) Direct intraosseous histoacryl injection. (c) Image after ending of histoacryl injection. Intraosseous injection of CMW3™ (d) in the posterior part of the lesion and (e) in the anterior part.

Digital subtraction angiograms clearly displayed the facial and its branches, inferior alveolar, and left lingual arteries (Figure 3(a)).

After this and under locoregional analgesia of the left mandible, direct histoacryl injection was completed with a low speed under fluoroscopy monitoring, in the angiographic suite through a percutaneous puncture performed with a 22-gauge puncture needle.

The volume of the histoacryl used varied around 4 to 5 ml (diluted 25% with lipiodol) (Figures 3(b) and 3(c)).

Then, bone cement with gentamicin (CMW3™) mixed with lipiodol was injected through the same 22-gauge puncture needle, first in the posterior part of the lesion (Figure 3(d)), followed by the anterior part (Figure 3(e)).

The percutaneous injections revealed abnormal venous lakes or pouch which depicts the venous lesion.

**Figure 4:**
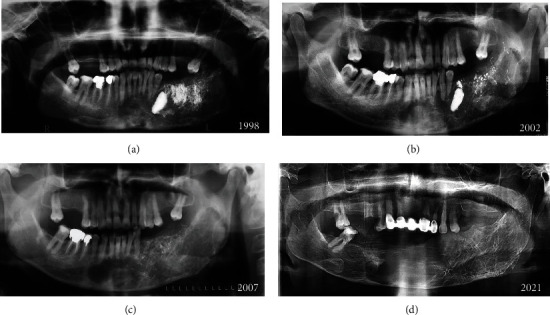
(a) The same day panoramic radiograph. (b) 4-year panoramic radiograph. (c) 9-year panoramic radiograph. (d) 22-year panoramic radiograph; note the calcification inside the lesion.

No complications were noted in the postoperative period, and the patient was discharged the same day. The patient was followed yearly from 1998 till 2020 (Figures 4(a)–4(d)).

The swelling was maintained with the same volume, and the only difficulties were losing her mandibular teeth.

**Figure 5:**
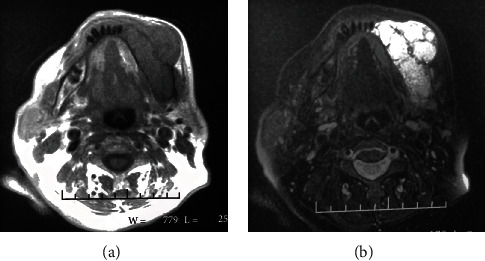
10-year MRI images: (a) after an axial T1WI MR image showing the stabilization of the old lesion; (b) an axial T2WI image presented the same hypersignals and expansible lesion.

Radiological images of 10-year follow-up MRI showed a stabilization of the lesion without any new extensions (Figures 5(a) and 5(b)).

## 3. Discussion

VMs are characterized by the irregular proliferation of endothelial cells of the blood vessels. They are considered as congenital inaccuracies of vascular morphogenesis that result in fragile endothelial cell proliferation inside the network of blood vessels [2].

VMs appear more frequently in soft tissue than in bone. In the mandible, they are very rare and represent only 0.2–1.0% of all intraosseous tumors, and the most common symptoms are pain, swelling, and tooth mobilities [5, 11]. Previous studies suggested that VMs appear more commonly in females than in males (ratio 3/1) [4]. Zlotogorski et al. reported that the molar region and angle of the mandible are affected in 69%, and the lesions were multilocular in 66% and unilocular in 33%, and 1% demonstrated a sunray radiographic shape [12].

Surgery is considered as the last treatment choice for AVMs of the mandible [4, 5]. Resection with block of the affected area in the mandible or maxilla has been proposed, and rehabilitation with autogenous or alloplastic bone materials has been recommended [13].

Endovascular therapy has been proposed for the treatment of the mandibular VMs with a success rate of 70%, and this evades mutilating surgery and related sequelae [4, 5]. Additionally, embolization can maintain functional anatomy and morphology [5].

Injection of emboli is suggested in a direct intraosseous technique; embolization should be achieved before to reduce the blood flow and to admit more precise accumulation of bone cement inside the lesion [7].

The biomaterials used as injectable embolic for the treatment of vascular lesions are intended to become occlusive by provoking thrombosis or directly obstructing the lesion when hardening of the embolic appears [14].

Different biomaterials have been used such as polymethylmethacrylate, n-butyl-2-cyanoacrylate, cellulose acetate polymer, fibrin glue, and bone cement [10].

In general, intraosseous VMs display hyposignals on T1WI and hypersignals on T2WI [15]. Slow-flow VMs are categorized by a varied intermediate signal without flow, voids on T1WI, and hypersignals on T2WI [15]. On T2WI, the lesions show well-defined borders and high contrast with the surroundings, with the lesion appearing as multispatial or multicystic [15].

The present case displayed homogenous hyposignals on T1WI and hypersignals on T2WI. The absence of signal voids advocates that we are facing a vascular malformation classified as a slow-flow lesion.

The MR images showed that the lesion was multilocular with bone cortical discontinuity with extension to the soft tissue.

The conservative treatment adopted a superselective angiographic embolization with the direct injection of histoacryl mixed with lipiodol and followed with the injection of bone cement in the two parts of the lesion. The first step reduces blood flow, and the second step is aimed at inducing thrombosis and at directly blocking the lesion.

The MR images 10 years later revealed stabilization of the size and extensions of the lesion. The panoramic radiograph after 22 years showed a bone formation inside the body of the mandible. The long follow-up period and the absence of any complications are favorable for the adopted treatment plan.

## 4. Conclusion

VMs need a very close multidisciplinary collaboration involving interventional radiology, otorhinolaryngology, oral/maxillofacial surgery, and plastic surgery in the arrangement and application of an appropriate treatment plan for these lesions.

## Data Availability

All available data are included in the manuscript.
